# Municipal police support for harm reduction services in officer-led referrals of people who inject drugs in Tijuana, Mexico

**DOI:** 10.1186/s12954-021-00513-4

**Published:** 2021-07-26

**Authors:** Pieter Baker, Jaime Arredondo, Annick Borquez, Erika Clairgue, Maria L. Mittal, Mario Morales, Teresita Rocha-Jimenez, Richard Garfein, Eyal Oren, Eileen Pitpitan, Steffanie A. Strathdee, Leo Beletsky, Javier A. Cepeda

**Affiliations:** 1 Department of Medicine, University of California San Diego School of Medicine, La Jolla, CA USA; 2 School of Public Health, San Diego State University, San Diego, CA USA; 3 Programa de Política de Drogas, Centro de Investigación Y Docencia Económica, Aguascalientes, Mexico; 4 British Columbia Centre on Substance Use, Vancouver, Canada; 5 School of Medicine, Universidad Xochicalco, Tijuana, Baja California México; 6 School of Government and Public Policy, University of Arizona, Tuscon, AZ USA; 7 Society and Health Research Center, Facultad de Humanidades, Universidad Mayor, Santiago, Chile; 8 School of Law & Bouvé College of Health Sciences, Northeastern University, Boston, MA USA; 9 Johns Hopkins Bloomberg School of Public Health, Baltimore, MD USA

**Keywords:** Police, Law enforcement, Syringe service program, Addiction, HIV, Referral, Harm reduction, People who use drugs

## Abstract

**Background:**

Police constitute a structural determinant of health and HIV risk of people who inject drugs (PWID), and negative encounters with law enforcement present significant barriers to PWID access to harm reduction services. Conversely, police may facilitate access via officer-led referrals, potentiating prevention of HIV, overdose, and drug-related harms. We aimed to identify police characteristics associated with support for officer-led referrals to addiction treatment services and syringe service programs (SSP). We hypothesized that officers who believe harm reduction services are contradictory to policing priorities in terms of safety and crime reduction will be less likely to support police referrals.

**Methods:**

Between January and June 2018, police officers (*n* = 305) in Tijuana, Mexico, completed self-administered surveys about referrals to harm reduction services during the 24-month follow-up visit as part of the SHIELD police training and longitudinal cohort study. Log-binomial regression was used to estimate adjusted prevalence ratios and model policing characteristics and attitudes related to officers’ support for including addiction treatment and SSP in referrals.

**Results:**

Respondents were primarily male (89%), patrol officers (86%) with a median age of 38 years (IQR 33–43). Overall, 89% endorsed referral to addiction services, whereas 53% endorsed SSP as acceptable targets of referrals. Officers endorsing addiction services were less likely to be assigned to high drug use districts (adjusted prevalence ratio [APR] = 0.50, 95% CI 0.24, 1.08) and more likely to agree that methadone programs reduce crime (APR = 4.66, 95% CI 2.05, 9.18) than officers who did not support addiction services. Officers endorsing SSPs were younger (adjusted prevalence ratio [APR] = 0.96 95% CI 0.93, 0.98), less likely to be assigned to high drug use districts (APR = 0.50, 95% CI 0.29, 0.87), more likely to believe that methadone programs reduce crime (APR = 2.43, 95% CI 1.30, 4.55), and less likely to believe that SSPs increase risk of needlestick injury for police (APR = 0.44, 0.27, 0.71).

**Conclusions:**

Beliefs related to the occupational impact of harm reduction services in terms of officer safety and crime reduction are associated with support for referral to related harm reduction services. Efforts to deflect PWID from carceral systems toward harm reduction by frontline police should include measures to improve officer knowledge and attitudes about harm reduction services as they relate to occupational safety and law enforcement priorities.

*Trial Registration*: NCT02444403.

## Background

Alongside rising global drug consumption patterns, drug-related harms such as overdose, Human Immunodeficiency Virus (HIV), and Hepatitis C virus (HCV) infection related to injection drug use (IDU) remain significant public health problems. North America has been particularly affected as unintended overdose is now recognized as the leading cause of accidental death in the USA [1, 2]. However, the global burden of disease due to opioid dependence is substantial [3]. There are an estimated 15.6 million people who inject drugs (PWID) worldwide, the global prevalence of HIV among PWID is 18%, and localized HIV outbreaks among PWID have been observed in numerous settings [4, 5]. Colliding syndemics of IDU, HIV, HCV and overdose have been further exacerbated by social and economic harms caused by the COVID-19 pandemic and mitigation strategies [6–9]. Resultant shifts in drug distribution and consumption patterns, in addition to augmented barriers to health and social services, make access to essential care for PWID a timely priority [6–9]. While the global burdens of substance use and related risk remain high, effective evidence-based public health interventions exist to reduce drug-related harms among people who inject drugs (PWID) [10].

Syringe service programs (SSP) are important public health interventions that are widely recognized to reduce the spread of bloodborne pathogens through IDU [10, 11]. Drug treatment paradigms vary greatly from abstinence-only programs to opioid agonist therapy (OAT) such as methadone maintenance therapy (MMT) and buprenorphine. In addition to reducing HIV risk through IDU cessation [10], retention in OAT is associated with reductions in all cause and unintended overdose mortality [12]. SSP and OAT represent effective and cost-effective harm reduction interventions to reduce the burden of drug-related harms among PWID [10–15]. However, PWID access to such services is precluded by significant barriers including cost [16–20], mobility [16, 21], migration/deportation [22, 23], stigma [16, 24], childcare/family needs [17], cultural and religious pressures [25], and police interference and/or harassment [26, 27].

The public health impact of policing has become increasingly recognized as a critical structural determinant of health, especially among PWID [28]. The harmful impact of incarceration on subsequent HIV risk has been well documented [29–31], but police also hold a significant role in the risk environment for HIV and drug-related harms outside the context of incarceration [32–34]. Abusive police–PWID interactions have been shown to drive HIV risk, risky injection behaviors, and harm reduction avoidance [28]. For example, police harassment, arrests and/or assaults outside of MMT or SSP sites may limit PWID willingness to utilize such sites [26–28, 35–37]. Given that prohibitive cost is already a barrier to accessing MMT for many PWID [20], being forced to pay a bribe to police may be particularly damaging to MMT utilization [27]. Police practices such as syringe confiscation may limit syringe access and discourage SSP utilization, leading to unsafe syringe sharing [28, 38, 39]. Additionally, in some settings, arrests may result in forced abstinence while in police custody or during coerced drug treatment, leading to an increased risk of overdose [40].

Tijuana, Mexico, provides an illustrative example of how drug law enforcement can be acutely harmful to the health of PWID populations. As a high-traffic border city, Tijuana is a nexus of drug trafficking, local drug consumption, and drug/sex tourism [17, 22, 41–45]. An estimated 12,000 PWID reside in Tijuana where HIV prevalence is approximately 4.2%, a burden of disease approximately ten times the national average [14]. Robust local research has described a blighted history of abusive drug law enforcement practices including large-scale police ‘crackdown’ operations, routine spatial regulation of homeless PWID, human rights abuses by police, forced drug detoxification, in addition to aggressive policing near harm reduction services [18, 27, 34, 35, 42, 46–51].

Police, as gatekeepers to the criminal justice system, also have the capacity to help deflect individuals in need of vital services to essential drug treatment and harm reduction services in lieu of arrest and incarceration. Due to frequent interactions with PWID, police behaviors can be leveraged to either cause public health harm or potentially deliver a positive public health impact. In referring PWID to evidence-based harm reduction services, police have the capacity to reduce drug-related harm. Additionally, as first responders, police may play a role in overdose reversal using naloxone [52]. Ideally, interventions to address drug law enforcement would serve to minimize the harms of abusive police practices while promoting positive outcomes stemming from police–PWID interaction (i.e., referrals).

While significant gaps in the literature remain on the topic of harm reduction training for police [53], educational programs targeting the interface between police and PWID have been successfully deployed to address public health harms caused by drug law enforcement. For example, the LEAD (Law Enforcement Assisted Diversion) program in Seattle, Washington, has demonstrated efficacy in diverting people into case management and supportive services in lieu of incarceration [52]. This analysis is rooted in the context of police training with the SHIELD (Safety and Health Integration in the Enforcement of Laws on Drugs) model that was implemented in Tijuana between 2015 and 2018. Details of the SHIELD training design and conceptual framework have been previously published (ClinicalTrials.gov Identifier: NCT02444403) [54]. In short, the intervention was designed using the Transcontextual Model (which incorporates elements of Theory of Planned Behavior and Social Determination Theory) to highlight and target pathways to behavioral change among police [54, 55]. During the training, officers received training on needlestick injury (NSI) prevention, HIV/HCV epidemiology and prevention, federal decriminalization reforms to drug policy, and elements of drug addiction and harm reduction strategies.

The SHIELD policing training model has been deployed in a number of settings and has demonstrated efficacy in improving police attitudes, knowledge, and intentions relevant for improving police–PWID interactions [56–60]. While the SHIELD training addresses the topic of harm reduction services, no officer-led referral programs exist in Tijuana and PWID are often forced into non-evidence-based drug treatment programs that may have negative consequences for some PWID, included unintended overdose after release [40]. Mixed methods research in Tijuana has identified moderate support for officer-involved referrals to harm reduction services among police and PWID alike [61]. Officer-held beliefs and attitudes regarding harm reduction services may shape, at least in part, their preference for including such services in a referral. However, there remains a gap in knowledge regarding relevant police characteristics and specific attitudes associated with referrals for harm reduction services.

The objective of this analysis was to evaluate police officer preferences for referrals of PWID to harm reduction services, including drug treatment and SSP, and to identify characteristics and attitudes associated with such preferences. We hypothesized that 1) officers that believe methadone programs reduce crime will be more likely to indicate addiction treatment services should be included in referrals and 2) officers that believe SSP increase the risk of needlestick injury will be less likely to indicate SSP should be included in referrals.

## Methods

### Study design

Between February 2015 and May 2016, 1808 active-duty municipal police officers in the Tijuana municipal police force were trained as part of an innovative police training utilizing the SHIELD model. All participants signed written informed consent and the study protocol was approved by the UCSD Human Research Protections Program (HRPP) and the Institutional Review Board of the Xochicalco University, Mexico.

### Data collection

Officers completed self-administered pre- and post-training surveys in Spanish and a subset of officers (*n* = 771) were randomly selected for 24-months of follow-up. These participants attended follow-up visits in the field or private settings convenient to the participant at 3, 6, 12, 18, and 24 months. We designed the questionnaire based on previous training interventions [20], adapted it for cultural considerations and clarity, piloted it alongside officers from the Tijuana Police Academy, and incorporated feedback. We collected data on socio-demographics, recent self-reported policing behaviors (e.g., syringe confiscation [last 6 months], physical altercation [last 6 months]) as well as current knowledge and attitudes related to drug policy, PWID, and drug addiction. Midway through the 24-month follow-up survey, and only at the 24-month visit, we administered a supplemental study which included additional survey items (analytical sample *n* = 305). These survey items covered police referral practices related to PWID, preferred services for referrals (including harm reduction services) and potential incentives for officers to facilitate referrals.

### Outcome measures

At the 24-month survey, officers were asked “Which services should be included in a referral” and responded either “Yes” or “No” to the following list of 11 individual referral services: drug/alcohol addiction services, syringe service programs, HIV or other infectious disease testing, HIV treatment, overdose prevention, wound care and other health care, dental clinic, food assistance, legal or immigration assistance, housing assistance, employment assistance, laundry, showers, or other personal care services. Our primary outcomes of interest for this analysis were officers’ preferences for referral to drug/alcohol addiction services or syringe service program services.

### Explanatory variables

To understand which factors were associated with officers’ preferences for harm reduction services inclusion in a referral, we also examined the following factors: self-efficacy to conduct a referral, perceived supervisory support, patrol assignment location (High drug use area vs. low drug use area) perceived role as a police officers and several attitudinal factors related to PWID, harm reduction services, and policing. The primary independent variables relevant for hypothesis testing were office-held beliefs related to the occupational impact of SSP and methadone. Beliefs regarding SSP were measured by the survey item “Syringe exchange programs increase the risk of NSI among police” and beliefs regarding methadone were measured by the survey item “Methadone maintenance programs help reduce criminal activity”. We measured these and all other explanatory variables on a 3-point Likert scale (Agree/Neutral/Disagree) and dichotomized them (Agree vs Neutral/Disagree) to distinguish between “positive” and “negative” perceptions of methadone and syringe service programs.

### Statistical analysis

In this cross-sectional analysis, we report descriptive statistics for the 24-month follow-up sample who completed the additional referral questionnaire. We excluded subjects with missing data for either of the outcomes (*n* = 8, 2.6%). We conducted bivariate analyses between all relevant factors and each of the two dependent variables (endorsing referral to drug addiction services and SSP). We then used log-binomial regression to estimate prevalence ratios and model policing characteristics and attitudes associated with officer support for including addiction treatment and SSP in referrals. To test our hypotheses, we created multivariable models in a forward, stepwise fashion. First, we introduced factors that were conceptually plausible and significantly associated with the outcome in bivariate analysis (p < 0.05) one by one from those with smaller to higher p-values. With the introduction of each factor into the model, we evaluated changes in Akaike information criterion (AIC) for each model and selected final models that minimized AIC. We report two adjusted models, one for each dependent variable (drug addiction services and SSP).

## Results

### Sample characteristics

A total of 305 officers were eligible for this analysis as they had completed the additional referral questionnaire at the 24-month study visit and had complete outcome data. The sample was predominantly male (89%), had at least a high school level of education (82%) and a median age of 38 years (Interquartile Range [IQR] = 33–43) (Table 1). As opposed to holding supervisory roles, most respondents held the rank of officer (86%) with a median of 12 years working on the force (IQR = 9–18) and 25% were assigned to high drug-use districts along the Tijuana River Canal (*n* = 77). Respondents reported high levels of referral self-efficacy (*n* = 282, 92%) and supervisory support (66%), and most perceived it was their role as police to refer PWID to health & social services (83%). As for attitudes related to harm reduction, 81% agreed that methadone maintenance programs helped reduce criminal activity while 51% disagreed that SSP increased the risk of NSI among police.

**Table 1:** Descriptive Characteristics of Municipal Police Officers in 24-month sample of SHIELD Cohort in Tijuana, Mexico (*n* = 305)

Police characteristics	n/median	%/IQR
Age (years)	38	33.0–43.0
# Years on Force	12	9.1–18.5
Gender		
Female	33	10.8
Male	272	89.1
Rank		
Officer	255	86.1
Supervisor/Deputy/Chief	50	16.4
Location		
High drug use district	77	25.3
Low drug use district	228	74.7
Education		
< High School	56	18.4
≥ High School	249	81.6
Police Support for Harm Reduction Referral Service		
Which service locations should be included in a police referral?		
Addiction services		
“Yes”	270	88.5
“No”	35	11.5
SSP		
“Yes”	161	52.8
“No”	144	47.2
Police attitudes		
My supervisor would commend me for referring PWID		
Agree	200	65.6
Neutral/Disagree	105	34.4
If I wanted to refer PWID to a health program, I would know how		
Agree	282	92.4
Neutral/Disagree	23	7.6
It is the role of police to refer PWID to health & social services		
Agree	252	82.6
Neutral/Disagree	53	17.4
Likelihood that PWID will go to service location if referred		
Always	85	27.9
Sometimes/Rarely/Never	220	72.1
Methadone maintenance programs help reduce criminal activity		
Agree	247	81.0
Neutral/Disagree	58	19.0
Syringe exchange programs increase the risk of NSI among police		
Agree	148	48.5
Neutral/Disagree	157	51.5
People addicted to drugs do not care about their health		
Agree	146	47.9
Neutral/Disagree	159	52.1
Drug Addiction is a disease		
Agree	283	92.8
Neutral/Disagree	22	7.2

### Referral to drug addiction services

Most respondents (86%) indicated that drug addiction services should be included in a police referral. In the unadjusted bivariate models (Table 2), officers assigned to high drug use districts were 54% (Prevalence Ratio [PR] = 0.46, 95% Confidence Interval [CI] 0.22, 0.95) less likely to support referral to drug addiction services than officers assigned to low drug use districts. Officers that agree methadone maintenance programs help reduce criminal activity were nearly five times more likely (PR = 4.55, CI 2.17, 9.56) to endorse referral to drug addiction services. In the adjusted model, the prevalence of indicating that drug addiction services should be included in a referral was 4.66 times higher (Adjusted Prevalence Ratio [APR] = 4.66, CI 2.05, 9.18) among officers who agreed that methadone maintenance programs help reduce criminal activity than those who did not agree, after controlling for district assignment location.

**Table 2:** Unadjusted and adjusted models of officers reporting which harm reduction services should be included in a referral (*n* = 305)

	Drug Addiction Treatment Services (“Yes”)				Syringe Service Programs (“Yes”)			
	Unadjusted model		Adjusted model		Unadjusted model		Adjusted model	
Police characteristics	Prevalence ratio	95% CI	Adjusted prevalence ratio	95% CI	Prevalence Ratio	95% CI	Adjusted Prevalence Ratio	95% CI
Age (years)	0.99	0.95, 1.03			0.98*	0.97, 0.99	0.96*	0.93, 0.98
# Years on Force	1.00	0.96, 1.05			0.98	0.97, 1.01		
Gender (Female vs Male)	0.54	0.21, 1.42			0.82	0.40, 1.69		
Rank (Officer vs Supervisor/Deputy/Chief)	0.75	0.29, 1.94			0.96	0.49, 1.85		
Location (high drug use vs Elsewhere)	0.46*	0.22, 0.95	0.5	0.24, 1.08	0.54*	0.32, 0.91	0.50*	0.29, 0.87
Education (< High School vs ≥ High School)	0.73	0.32, 1.71			0.61	0.34, 1.09		
Attitudes (Agree vs Neutral/Disagree)								
My supervisor would commend me for referring PWID	1.56	0.56, 2.40			0.84	0.52, 1.36		
If I wanted to refer PWID to a health program, I would know how	0.33	0.04, 2.53			0.86	0.36, 2.02		
It is the role of police to refer PWID to health & social services	1.77	0.78, 4.04			1.59	0.88, 2.90		
Likelihood that PWID will go to service location if referred	0.81	0.37, 1.74			1.01	0.61, 1.68		
Methadone maintenance programs help reduce criminal activity	4.55*	2.17, 9.56	4.66*	2.05, 9.18	2.33*	1.29, 4.21	2.43*	1.30, 4.55
Syringe exchange programs increase the risk of NSI among police	0.78	0.38, 1.57			0.46*	0.29, 0.73	0.44*	0.27, 0.71
People addicted to drugs do not care about their health	0.65	0.32, 1.33			1.12	0.72, 1.76		
Drug Addiction is a disease	1.23	0.35, 4.40			1.38	0.58, 3.31		

*Significant at alpha < 0.05

### Referral to SSP services

More than half of the respondents indicated that SSP services should be included in a referral (53%). In the bivariate models (Table 2), support for referral to SSP services was significantly associated with age (PR = 0.98, CI = 0.97, 0.99), district assignment location (PR = 0.54, CI = 0.32, 0.91), agreeing that methadone maintenance programs help reduce criminal activity (PR = 2.33, CI = 1.29, 4.21) and agreeing that SSPs *increase* the risk of NSI among police (PR = 0.46, CI = 0.29, 0.73). In the adjusted model, the prevalence of indicating that SSP services should be included in a referral was 4% lower for each 1 year increase in age (APR = 0.96 per year, CI = 0.93, 0.98), 50% lower among officers assigned to high drug use districts along the Tijuana River Canal (APR = 0.50, CI = 0.29, 0.87), 2.43 times higher (APR = 2.43, CI = 1.30, 4.55) among officers who agreed that methadone maintenance programs help reduce criminal activity, and 56% lower among those who agreed that SSPs increase the risk of NSI among police (APR = 0.44, CI = 0.27, 0.71).

## Discussion

Most officers indicated that drug addiction services should be included in a police referral while about half indicated SSP services. Preference for drug addiction services was associated with the belief that MMT programs help reduce criminal activity. Preference for SSP services was associated with age, assignment to high drug use districts along the Tijuana River Canal and positive attitudes regarding the occupational impact of MMT and SSP. These findings support our hypothesis that police support for SSP and drug addiction treatment services are associated with officer-held beliefs regarding the occupational impact of harm reduction services (i.e., MMT and SSP).

Our findings suggest that the perceived occupational impact of harm reduction programs may shape, at least in part, officer willingness to refer PWID to certain programs. Its logical that police, as with workers in any occupation, would support programs they perceive to make their job easier (reduce crime) and reject those they perceive as unsafe (increase risk of NSI). Further, it is logical to hypothesize that these occupational beliefs about such services may also shape officer behavior around harm reduction sites and their clientele. Recent qualitative research among police has suggested that educating police about harm reduction operations such as safe consumption sites could improve relationships between police and harm reduction programs [62]. Highlighting the occupational benefits of harm reduction programs to police should be prioritized in efforts to align public health and police work, including training and officer-led referral programs.

Officers assigned to high drug use districts along the Tijuana River canal were less likely to indicate that drug treatment or SSP services should be included in a referral. This is consistent with previous research in Tijuana suggesting police attitudes and behaviors toward PWID among officers assigned to these districts may be more negative than those of officers assigned to low drug use districts [48]. Officers assigned to such areas in Tijuana were more likely to arrest PWID for syringe possession, arrest for heroin, possession and confiscate syringes [21, 22]. Previous geospatial research has identified hotspots of self-reported arrest for any offense, police stops, and extrajudicial police encounters in these areas of Tijuana [17, 18]. It is possible that lived experience working in high drug use areas may alter officer perceptions of PWID, addiction, and harm reductions services differently than officers assigned to lower drug use areas. Police burnout may play an outsized role among police assigned to these areas, leading to more pessimistic attitudes than their counterparts in spaces with less prevalent drug use [63, 64]. Policing in these areas may also present an elevated risk of occupational hazards such as needlestick injury, a factor associated with harmful police practices like syringe confiscation and negative attitudes toward harm reduction services such as SSP [64]. Alternatively, it could be the case that officers are specifically selected for assignment to high drug areas because of these existing characteristics and attitudes. Notably, before the training, officers assigned to the *Zona* Centro district (a high drug use neighborhood) were more likely to refer PWID to health or social programs than officers assigned elsewhere [22]. This may be due to a higher number of opportunities to refer given the clustering of PWID and drug treatment centers in this area [19, 20].

Previous research with this cohort has demonstrated that these attitudes toward PWID are not necessarily associated with an increased likelihood of referral behavior [22]. Qualitative research has examined structural barriers to referrals such as perceived dysfunction of drug treatment centers and fear of resentment from PWID [47]. In this analysis, none of the attitudes specifically related to referral self-efficacy, supervisory support or perceived role to refer were associated with either outcome. Using baseline data (pre-training) in this cohort, we had previously demonstrated that officers who agreed it was their role to refer PWID to health and social services were 3.32 times more likely to refer PWID in the last 6 months [22]. It may be that attitudes related to their role influence referral behaviors among police, but do not influence which services officers perceive should be included in a referral. Also, it may be difficult to distinguish between voluntary referrals (as specified in the referral survey completed for this analysis) and coerced referrals in this context as forced admissions to drug treatment, typically abstinence-based programs, is common [40, 47, 65].

Research in other settings has successfully demonstrated that police referral programs may be feasible to implement with a degree of acceptability among PWID [66]. However, fragmented treatment systems remain a barrier to long-term recovery among participants. Police referrals alone, without scale-up and coordination with evidence-based harm reduction interventions, may result in coerced admission to detoxification and/or abstinence-based programs. In such instances, police referrals constitute an additional source of damage as coerced detox “treatment” paradigms have been associated with harm among PWID, including higher likelihood of experiencing non-fatal overdose [40, 67].

This research is relevant to arguments supporting the deflection of police responsibilities and power in favor of more effective and cost-effective interventions to address drug-related harms and PWID. Our findings suggest that police willingness to refer PWID to harm reduction services depends on police characteristics (assignment location, occupation-related attitudes toward harm reduction services). These are factors which may be heavily shaped by law enforcement institutional norms; therefore, efforts to increase referrals in this context must promote correct understandings and more positive perceptions of harm reduction services. However, this issue also supports arguments outside the scope of this analysis which suggest that law enforcement personnel are not ideal candidates for street-level interventions with PWID, including referrals. Given police officers’ carceral legal tools and vocational norms, the role of referral to harm reduction services may best be carried out by actors and systems of support (including integrated service facilities) alternative to law enforcement. Considering the known harms of street drug law enforcement practices, including the potential for harmful and/or coerced police referrals, the role of police in drug treatment referral remains somewhat precarious. We suggest the following twofold strategy to mitigate the public health harms of drug law enforcement with regard to harm reduction: (1) decrease routine interaction between PWID and police when possible and (2) shift PWID–police interactions from a source of potential harm (i.e., arrest leading to incarceration) to that of assistance. Drug policy reform, coupled with interventions like the SHIELD training which target police knowledge, attitudes and behaviors, may be necessary to achieve these aims and warrant further examination.

There are several limitations for this study. Since the sample consisted of officers in the SHIELD cohort who had been exposed to an educational intervention, the results may not be generalizable to officers that have not received relevant police training or officers working in other settings. Also, some officers may not have differentiated between drug addiction and alcohol addiction services in selected referral service preferences. There is potential for social desirability bias if officers responded to the surveys in a way to be perceived favorably by the study staff; however, self-administered surveys were implemented to reduce such bias. Finally, as a cross-sectional study with hypothetical outcomes, no inferences regarding causality or referral behavior can be made.

## Conclusions

Officers’ willingness to indicate drug treatment services in referrals was associated with positive attitudes toward MMT, whereas willingness to indicate SSP was associated with age, patrol location, and positive attitudes toward MMT and SSP programs. Referrals to evidence-based harm reduction services carry potential to reduce drug-related harms among PWID but may rely on shifting police perspectives. Interventions designed to improve PWID–police interactions such as police trainings should target officer beliefs and attitudes toward harm reduction services. Positive perceptions of harm reduction services must be promoted alongside the expansion of evidence-based services.

## Data Availability

The datasets used and/or analyzed during the current study are available from the corresponding author upon reasonable request.

## Abbreviations

APR: Adjusted prevalence ratio
CI: Confidence interval
HIV: Human immunodeficiency virus
IDU: Injection drug use
MMT: Methadone maintenance therapy
NSI: Needlestick injury
OAT: Opioid agonist therapy
PWID: People who inject drugs
SSP: Syringe service program
SHIELD: Safety and Health Integration in the Enforcement of Laws on Drugs
